# Traditional Chinese Exercises on Pain and Disability in Middle-Aged and Elderly Patients With Neck Pain: A Systematic Review and Meta-Analysis of Randomized Controlled Trials

**DOI:** 10.3389/fnagi.2022.912945

**Published:** 2022-06-10

**Authors:** Lingjun Kong, Jun Ren, Sitong Fang, Tianxiang He, Xin Zhou, Min Fang

**Affiliations:** ^1^Yueyang Hospital of Integrated Traditional Chinese and Western Medicine, Shanghai University of Traditional Chinese Medicine, Shanghai, China; ^2^Institute of Tuina, Shanghai Institute of Traditional Chinese Medicine, Shanghai, China; ^3^Shuguang Hospital, Shanghai University of Traditional Chinese Medicine, Shanghai, China

**Keywords:** traditional Chinese exercises, neck pain, disability, meta-analysis, complementary and alternative therapy

## Abstract

**Background:**

With the change of life and work style, more middle-aged and elderly individuals are suffering from neck pain. In China, traditional Chinese exercises (TCEs) are widely used in the management of neck pain, such as Tai Chi, Qigong, Yijinjing, Baduanjin, Liuzijue, and Five-animal exercises. However, the evidence of TCEs for neck pain maintains controversial. Therefore, the current systematic review was conducted to evaluate the effects of TCEs on pain and disability of middle-aged and elderly patients with neck pain.

**Methods:**

A comprehensive literature search was performed in six electronic databases from their inception to January 2022 for randomized controlled trials of TCEs for neck pain. The methodological quality of the included studies was assessed by PEDro scale. The subgroup analysis was conducted based on different TCEs. The *I*^2^ statistic was applied to assess the heterogeneity.

**Results:**

Twenty-one studies were included in our review, which were conducted in China, United States, and Germany between 2003 and 2021. Most (86%) of them exceeded the cut off score 6. TCEs included Baduanjin, Yijinjing, Tai Chi, Qigong, and Five-animal exercises. The aggregated results indicated that TCEs showed positive complementary effects in relieving pain (*SMD*, 1.12; 95% *CI*, 0.78–1.45; *p* < 0.00001), especially Baduanjin exercises. Baduanjin exercises also showed beneficial complementary effects in improving flexion (*SMD*, 0.65; 95% *CI*, 0.28–1.03; *p* = 0.0006) and extension (*SMD*, 0.66; 95% *CI*, 0.12–1.19; *p* = 0.02) of the neck. In addition, the aggregated results indicated that TCEs alone showed beneficial effects in improving disability (*SMD*, 0.74; 95% *CI*, 0.40–1.08; *p* < 0.0001) and relieving pain (*SMD*, 0.81; 95% *CI*, 0.50–1.13; *p* < 0.00001) compared with waiting list. The follow-up effects of TCEs were still insufficient.

**Conclusion:**

There was the positive evidence to support the clinical use of TCEs, as a complementary therapy, for middle-aged and elderly patients with neck pain, especially Baduanjin exercises. However, the evidence supporting the effects of TCEs alone for the middle-aged and elderly patients with neck pain was limited due to the small sample size.

**Systematic Review Registration:**

https://inplasy.com/inplasy-2022-4-0083/, identifier INPLASY202240083.

## Introduction

Neck pain is a common musculoskeletal condition with a high incidence rate, and it is the fourth leading cause of years lived with a disability around the world (Vos et al., 2012). The estimated annual incidence of neck pain ranged between 10.4 and 21.3% (Hoy et al., 2010), with a higher incidence in middle-aged office workers and elderly population. In China, the incidence rate of neck pain was up to 24.66%, and it increases with age. The prevalence of neck pain was 34.94% among people over 60 years old (Xu, 2019). More than 60% patients with neck pain may experience the second neck pain within 1 year (Carroll et al., 2008). It places a significant burden on the patients with neck pain and social healthcare systems due to the treatment costs, work absenteeism, and reduced productivity (Cote et al., 2013). The study reported that 14.2% patients with neck pain have multiple episodes of work absenteeism within 2 years of their initial health claims due to neck pain (Van Eerd et al., 2011).

Health professionals and patients still face tremendous challenges on the management of neck pain due to a variety of treatment strategies and conflicting reports on its treatments (Cohen, 2015). Considering drug dependence and poor stability of the spine after neck surgery, more patients turn to choose complementary and alternative therapies for neck pain to relieve pain and improve quality of life. Exercises have been recommended to prevent and treat neck pain by the clinical guidelines (Bussières et al., 2016). Common exercise therapies for neck pain include range-of-motion exercise, aerobic exercise, supervised exercise, strengthening exercise, and yoga, which can possibly alleviate neck pain because of their ability to improve muscle strength, flexibility, and endurance as well as restore injured tissues (Jordan et al., 1997; Wolsko et al., 2003; Kose et al., 2007).

In China, traditional Chinese exercises (TCEs) are widely used in the treatment of neck pain and associated conditions, which include Tai Chi, Qigong, Yijinjing, Baduanjin, Liuzijue, and Five-animal exercises (Xie et al., 2021). TCEs take both body and mind into account, emphasize the coordination and unification of breathing and body movements under the guidance of consciousness, and exercise the muscles and joints of the whole body (Lan et al., 2013; Solloway et al., 2016). They may also improve balance, aerobic capacity, and regulate the cervical balance of dynamic and static mechanics, so as to improve neck function, control posture, and relieve pain (Kuo et al., 2021). The study also reported that the movements of TCEs are thought to relax the mind and body to dilate blood vessels and promote local blood circulation (Huston and McFarlane, 2016). As one of low to moderate-intensity mind-body exercises, TCEs may have similar health benefits with common exercise therapy, but experience lower energy metabolism. These traditional mind-body exercises are suitable for middle-aged and elderly patients with neck pain and mental health conditions. Some studies (von Trott et al., 2009; Lauche et al., 2016; Huang et al., 2020) reported the positive effects of TCEs in the management of neck pain. However, the previous review (Xie et al., 2021) showed that there was insufficient evidence to support the effects of TCEs in improving pain intensity and enhancing functional mobility in individuals with neck pain. The evidence of TCEs for neck pain maintains controversial.

Therefore, the current systematic review evaluated the effects of TCEs on pain and disability in middle-aged and elderly patients with neck pain. It provided evidence-based information for the clinical application of TCEs for neck pain.

## Methods

The systematic review was registered on the international platform of registered systematic review and meta-analysis. The registration number is INPLASY202240083.

### Search Strategy

A computerized literature search was conducted to identify the potential eligible studies in the following electronic databases from their inceptions to January 2022: PubMed, EMBASE, Web of Science, China National Knowledge Infrastructure (CNKI), Wanfang Degree and Conference Papers Database, and Chinese Science and Technology Periodical (VIP) Databases. The key searching terms were (“traditional Chinese exercises” OR “Tai Chi” OR “tai ji” OR “Baduanjin” OR “Yijinjing” OR “Qigong” OR “Liuzijue” OR “Five-animal exercises” OR “Wuqinxi”) and (“neck pain” OR “cervical spondylopathy” OR “cervical pain”). Additional studies were identified by scanning the reference list of relevant reviews. The World Health Organization International Clinical Trials Registry Platform (ICTRP) and the Chinese Clinical Trial Registry (ChiCTR) were searched to identify the ongoing or unpublished studies. When necessary, the reviewers contacted the study authors. There were no restrictions on publication language or status.

### Study Selection

According to the inclusion and exclusion criteria, the literature selection was performed by two reviewers independently. In this review, the inclusion and exclusion criteria were: (1) Types of study: randomized control trials (RCTs). (2) Types of participants: participants with a clinical diagnosis of neck pain, and the average age more than 40 years at least one group. There were no limitations on gender or nationality. (3) Types of interventions: TCEs were Tai Chi, Baduanjin, Yijinjing, Qigong, Liuzijue, and Five-animal exercises. The control interventions included waiting list, education, routine rehabilitation therapy, acupuncture, medicine, other modern exercise therapy, and any treatments without TCEs. (4) Types of outcomes: pain was measured by the visual analogue scale (VAS), functional mobility of the neck was assessed using the neck disability index (NDI), quality of life was assessed using the 36-item short form health survey (SF-36), and cervical range of motion such as flexion, extension, lateral-flexion, and rotation.

Trials that met any of the following criteria were excluded: (1) quasi-randomized RCTs and non-randomized trials, (2) duplicated publications, and (3) unavailable full text or missing data. Any disagreements were resolved by discussion between two reviewers.

### Data Extraction

The data extraction was independently performed by two reviewers based on pre-defined criteria. The following data were collected: (1) basic information such as the name of the first author, year, and country of publication; (2) characteristics of the participants such as sample size and mean age; (3) information regarding study design such as interventions, outcomes, and follow-up duration. Only the first phase data were extracted in the crossover studies. Any disagreements were resolved by discussion between two reviewers.

### Quality Assessment

Two reviewers independently assessed the methodological quality of the included trials by the Physiotherapy Evidence Database (PEDro) scale. The PEDro scale is a multi-item scale consisting of 11 items to measure the methodological quality of RCTs of physiotherapy interventions. The PEDro scale includes: (1) study eligibility criteria specified, (2) random allocation of subjects, (3) concealed allocation, (4) measure of similarity between groups at baseline, (5) subject blinding, (6) therapist blinding, (7) assessor blinding, (8) less than 15% dropouts, (9) intention-to-treat analysis, (10) between-group statistical comparisons, and (11) point measures and variability data. The PEDro score of 0–10 is obtained by summation (item 1 is not scored), and a cut point of 6 indicates high-quality studies. The PEDro score has been demonstrated to be valid and reliable for assessing methodological quality of physiotherapy RCTs (Maher et al., 2003; de Morton, 2009). Any disagreements were resolved by obtaining the consensus of all reviewers.

### Data Synthesis and Analysis

The meta-analysis was conducted using the RevMan version 5.3 (The Cochrane Collaboration, Software Update, Oxford, United Kingdom). For the continuous outcomes, the between-groups mean differences of the studies were converted to the standardized mean difference (*SMD*) with 95% confidence intervals (*CIs*) in the meta-analysis. A random effects model was used for better dealing with the clinical heterogeneity. The heterogeneity was evaluated using the *I*^2^ statistic: *I*^2^ > 30% suggests moderate heterogeneity, *I*^2^ > 50% represents substantial heterogeneity, and *I*^2^ > 75% considerable heterogeneity. The subgroup analysis was conducted based on different TCEs and control interventions. The risk of publication bias was assessed by funnel-plot if more than nine trials were included in the meta-analysis. Results were considered statistically significant for *p* < 0.05.

## Results

### Search and Selection

**FIGURE 1:**
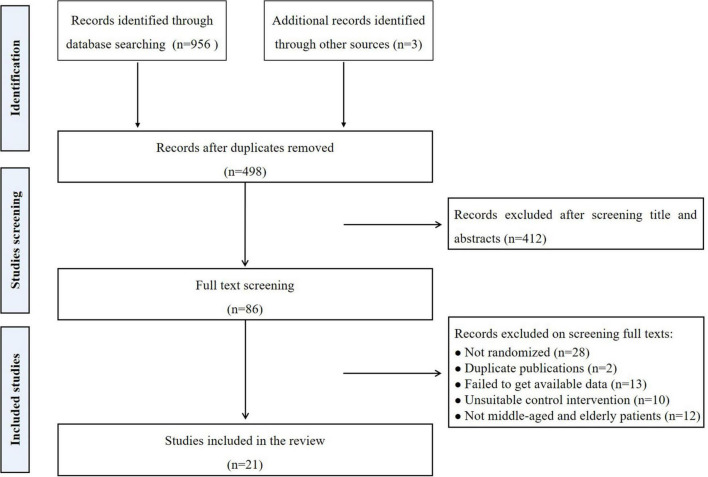
Flow chart for the review.

In our initial search, a total of 959 articles were found. A total of 461 of them were duplicates and thus excluded. A total of 412 studies were excluded after screening the titles and abstracts. After screening the full texts, 21 studies were included in our review. The detailed process was showen in Figure 1.

### Characteristics of the Included Studies

A total of 1,770 participants with a mean age of 49.59 ± 9.27 years were included in eligible 21 RCTs, which were conducted in China, United States, and Germany from 2003 to 2021. Sixteen studies used TCEs as a complementary therapy for neck pain. Five studies used TCEs alone for neck pain. In the included studies, TCEs included Baduanjin, Yijinjing, Tai Chi, Qigong, and Five-animal exercises. TCEs were combined with acupuncture, Tuina, routine rehabilitation, and medicine for neck pain in the included studies. The exercise duration was between 20 days and 24 weeks. The time of each exercise was between 5 and 90 min. The exercise ranged from 12 to 240 sessions. The follow-up time ranged from 12 to 24 weeks. The control intervention included waiting list, education, traction, acupuncture, Tuina, routine rehabilitation, medicine, and other exercises. The main outcomes included neck pain, neck disability, quality of life, and cervical range of motion. The main characteristics of all included RCTs were shown in Table 1.

**TABLE 1:** Randomized controlled trials evaluating the effects of traditional Chinese exercises for neck pain.

Study	Sample size	Mean age (years)	Duration (weeks)	Follow-up (weeks)	Main outcomes	Experimental group intervention	Control group intervention
**Traditional Chinese exercises alone for neck pain**							
von Trott et al. (2009), United States	38 39 40	75.9 ± 7.6 76.0 ± 7.2 75.7 ± 7.6	12	24	VAS, NPAD, ADS, SF-36	Qigong (45 min/session, 24 sessions, 12 weeks)	(1) Exercise (45 min/session, 24 sessions, 12 weeks) (2) Waiting list
Rendant et al. (2011), United States	42 39 41	44.7 ± 10.8 44.4 ± 10.9 47.8 ± 10.3	24	/	VAS, NPAD, SF-36	Tai Chi (18 sessions, 24 weeks)	(1) Exercise (18 sessions, 24 weeks) (2) Waiting list
Lin (2015), China	25 25	40.33 ± 7.33 39.98 ± 8.34	10	24	VAS, NPQ, ROM	Traditional Chinese exercises (80 min/session, 50–60 sessions, 10 weeks)	Tuina (60 min/session, 40 sessions, 10 weeks)
Lauche et al. (2016), Germany	38 39 37	52.0 ± 10.9 49.2 ± 11.7 47.0 ± 12.3	12	24	VAS, NDI, SF-36, HADS FEW-16, PSS, PAS, MAIA	Tai Chi (75–90 min/session, 12 sessions, 12 weeks)	(1) Exercise (60–75 min/week, 12 sessions, 12 weeks) (2) Waiting list
Zhou et al. (2017), China	32 33	59.71 ± 4.84 60.12 ± 4.56	10	/	NDI	Five-animal exercises (40 min/session, 30 sessions, 10 weeks)	Traction (20 min/session, 30 sessions, 10 weeks)
**Traditional Chinese exercises as complementary therapy for neck pain**							
Xiao et al. (2003), China	26 18	52 50	30 days	/	VAS	Traditional Chinese exercises (30–40 min/session, 60 sessions, 30 days) plus Chinese herb (2 sessions/days, 60 sessions, 30 days)	Chinese herb (2 sessions/days, 60 sessions, 30 days)
He (2014), China	30 30	43.23 ± 8.01 41.5 ± 7.45	12	/	VAS, NDI, SAS, SDS	Baduanjin (50–60 min/session, 84 sessions, 12 weeks) plus Tuina (40 min/session, 10 sessions, 2 weeks)	Tuina (40 min/session, 10 sessions, 2 weeks)
Hu et al. (2014), China	125 125	44.55 ± 12.42 45.02 ± 12.2	24	/	VAS, NDI, SF-36	Traditional Chinese exercises (40 min/session, 12 sessions, 12 weeks)	Waiting-list
Cai et al. (2015), China	30 30	50.8 ± 8.0 49.9 ± 8.0	60 days	/	VAS	Baduanjin (30 min/session, 120 sessions, 60 days) plus medicine, traction, and physical therapy (15–20 min/session, 120 sessions, 60 days)	Medicine, traction, and physical therapy (15–20 min/session, 120 sessions, 60 days)
Feng and Qin (2017), China	50 50 50	45.55 ± 7.33 50.22 ± 4.68 48.67 ± 6.56	4	12	VAS	Baduanjin (20 min/session, 48 sessions, 4 weeks) plus massage (20 min/session, 24 sessions, 4 weeks)	(1) Massage (20 min/session, 24 sessions, 4 weeks) (2) Massage (20 min/session, 24 sessions, 4 weeks) plus exercise (10 min/session, 48 sessions, 4 weeks)
Cai et al. (2018), China	36 33	57.49 ± 6.13 55.26 ± 4.75	24	/	NPQ, NPRS, PCS, MCS	Baduanjin (30 min/session, 240 sessions, 24 weeks) plus education (2 sessions, 24 weeks)	Education (2 sessions, 24 weeks)
Zheng (2018), China	30 30	45.32 ± 6.11 46.19 ± 6.39	12	/	VAS, ROM	Yijinjing (20 min/session, 144 sessions, 12 weeks) plus acupuncture (6–12 sessions)	Acupuncture (6–12 sessions)
Jiang et al. (2019), China	30 30	42.87 ± 8.80 44.00 ± 9.54	20 days	/	Cervical Arc Chord Distances	Five-animals exercise (2 sessions/day, 40 sessions, 20 days) plus routine rehabilitation (25 min/session, 20 sessions, 20 days)	Routine rehabilitation (25 min/session, 20 sessions, 20 days)
Wu et al. (2019), China	35 33	57.80 ± 5.61 56.30 ± 5.32	8	/	NDI, ROM, VAS	Baduanjin (30–40 min/session, 3–5 sessions/week, 8 weeks) plus conventional treatment (8 weeks)	Conventional treatment (8 weeks)
Chen et al. (2020), China	48 46	38.7 ± 8.4 40.5 ± 8.6	12	/	VAS, PPI	Qigong (30 min/session, 60 sessions, 12 weeks) plus acupuncture (4 weeks)	Acupuncture (4 weeks)
Huang et al. (2020), China	24 24	38.97 ± 2.37 40.26 ± 2.09	8	/	VAS, ROM	Baduanjin (10 min/session, 144 sessions, 8 weeks) plus Tuina (20 min/session, 24 sessions, 8 weeks)	Tuina (20 min/session, 24 sessions, 8 weeks)
Xie (2020), China	42 42	56.34 ± 6.45 55.98 ± 6.71	12	/	NPQ, MPQ	Baduanjin (5–8 sessions/day, 12 weeks) plus acupuncture (30 min/session, 24 sessions, 12 weeks) and medicine	Acupuncture (30 min/session, 24 sessions, 12 weeks) and medicine
Chen (2021), China	30 30	39.27 ± 9.40 40.00 ± 7.89	4	/	VAS, NDI, ROM	Baduanjin (12 sessions, 4 weeks) plus electroacupuncture (20 min/session, 12 sessions, 4 weeks)	Electroacupuncture (20 min/session, 12 sessions, 4 weeks)
Xu et al. (2021), China	30 30	54.93 ± 11.21 55.27 ± 9.84	4	/	VAS	Baduanjin (56 sessions, 4 weeks) plus acupuncture (20 min/session, 12 sessions, 4 weeks)	Acupuncture (20 min/session, 12 sessions, 4 weeks)
Yang et al. (2021), China	28 27	49.62 ± 7.83 46.45 ± 9.91	2	/	VAS, HAMA	Baduanjin (5 min/session, 42 sessions, 2 weeks) plus physiotherapy and education	Physiotherapy and education
Zhang (2021), China	40 40	43.21 ± 6.24 42.42 ± 6.25	/	/	VAS, PSQI	Baduanjin (40–60 min/session) plus routine rehabilitation	Routine rehabilitation

*ADS, Allgemeine Depressions-skala; FEW-16, Questionnaire for Assessing Subjective Physical Well-being; HADS, Hospital Anxiety and Depression Scale; HAMA, Hamilton Anxiety Scale; PCS, Physical Component Summary; ROM, Range of Motion; MAIA, Multidimensional Assessment of Interoceptive Awareness Instrument; MCS, Mental Component Summary; MPQ, McGill Pain Questionnaire; NDI, Neck Disability Index; NPAD, Neck Pain and Disability Scale; NPQ, Northwick Park Neck Pain Questionnaire; NPRS, Numerical Pain Rating Scale; PAS, Postural Awareness Scale; PPI, Present Pain Intensity; PSS, German Version of the Perceived Stress Scale; PSQI, Pittsburgh Sleep Quality Index; SAS, Self-Rating Anxiety Scale; SDS, Self-rating Depression Scale; SF-36, 36-Item Short Form Health Survey; VAS, Visual Analogue Scale.*

### Methodological Quality

Based on the PEDro scale, most (86%) of the included studies have good methodological quality with the scores ranging from 6 to 7 points. The most common flaws were blinding methods in these studies. Blinding of subjects and therapies were absent in all included RCTs. Only 2 RCTs describe concealed allocation in details (von Trott et al., 2009; Lauche et al., 2016). The assessor blinding was performed in 4 included studies (Hu et al., 2014; Lauche et al., 2016; Cai et al., 2018; Yang et al., 2021). In addition, four trials rated the intention-to-treat negative (Hu et al., 2014; Feng and Qin, 2017; Zhou et al., 2017; Huang et al., 2020), and three studies showed > 15% dropouts (Lauche et al., 2016; Feng and Qin, 2017; Huang et al., 2020). Other items were scored positive in the included studies. The detailed scores were shown in Table 2.

**TABLE 2:** PEDro scale of quality for the included studies.

Study	Eligibility criteria	Random allocation	Concealed allocation	Similar at baseline	Subjects blinded	Therapists blinded	Assessors blinded	< 15% dropouts	Intention-to-treat analysis	Between-group comparisons	Point measures and variability data	Total
**Traditional Chinese exercises alone for neck pain**												
von Trott et al. (2009)	1	1	1	1	0	0	0	1	1	1	1	7
Rendant et al. (2011)	1	1	0	1	0	0	0	1	1	1	1	6
Lin (2015)	1	1	0	1	0	0	0	1	1	1	1	6
Lauche et al. (2016)	1	1	1	1	0	0	1	0	1	1	1	7
Zhou et al. (2017)	1	1	0	1	0	0	0	1	0	1	1	5
**Traditional Chinese exercises as complementary therapy for neck pain**												
Xiao et al. (2003)	1	1	0	1	0	0	0	1	1	1	1	6
He (2014)	1	1	0	1	0	0	0	1	1	1	1	6
Hu et al. (2014)	1	1	0	1	0	0	1	1	0	1	1	6
Cai et al. (2015)	1	1	0	1	0	0	0	1	1	1	1	6
Feng and Qin (2017)	1	1	0	1	0	0	0	0	0	1	1	4
Cai et al. (2018)	1	1	0	1	0	0	1	1	1	1	1	7
Zheng (2018)	1	1	0	1	0	0	0	1	1	1	1	6
Jiang et al. (2019)	1	1	0	1	0	0	0	1	1	1	1	6
Wu et al. (2019)	1	1	0	1	0	0	0	1	1	1	1	6
Chen et al. (2020)	1	1	0	1	0	0	0	1	1	1	1	6
Huang et al. (2020)	1	1	0	1	0	0	0	0	0	1	1	4
Xie (2020)	1	1	0	1	0	0	0	1	1	1	1	6
Chen (2021)	1	1	0	1	0	0	0	1	1	1	1	6
Xu et al. (2021)	1	1	0	1	0	0	0	1	1	1	1	6
Yang et al. (2021)	1	1	0	1	0	0	1	1	1	1	1	7
Zhang (2021)	1	1	0	1	0	0	0	1	1	1	1	6

*Criteria (2–11) were used to calculate the total PEDro score. Each criterion was scored as either 1 or 0 according to whether the criteria was met or not, respectively.*

### Pain

**FIGURE 2:**
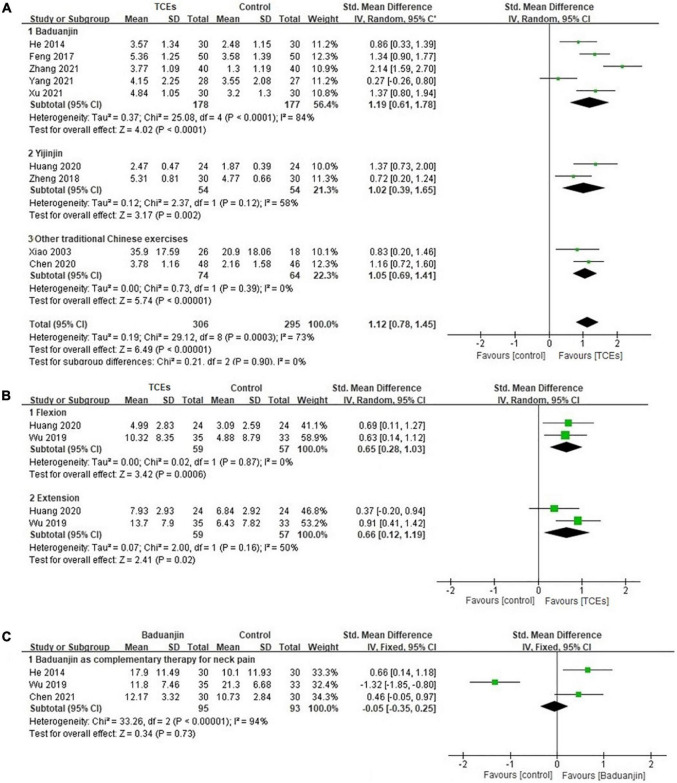
Forest plot of the complementary effects of traditional Chinese exercises (TCEs) for neck pain: **(A)** Pain, **(B)** range of motion, and **(C)** disability.

As a complementary therapy, fifteen studies reported the complementary effects in relieving pain of TCEs for neck pain. Nine studies were included in the meta-analysis. The aggregated results indicated that the TCEs showed positive complementary effects in relieving pain (*SMD*, 1.12; 95% *CI*, 0.78–1.45; *p* < 0.00001, Figure 2A), especially Baduanjin (*SMD*, 1.19; 95% *CI*, 0.61–1.78; *p* < 0.0001, Figure 2A) and Yijinjing (*SMD*, 1.02; 95% *CI*, 0.39–1.65; *p* = 0.002, Figure 2A).

**FIGURE 3:**
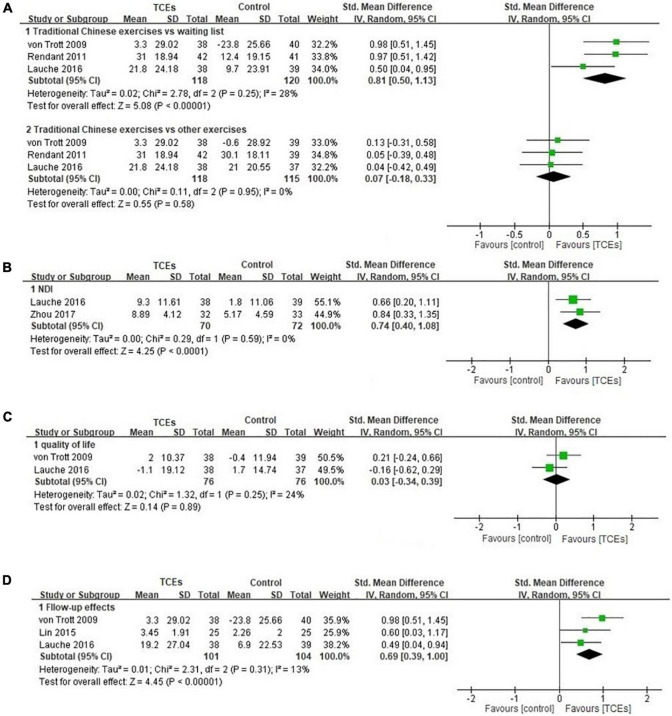
Forest plot of the effects of traditional Chinese exercises (TCEs) alone for neck pain: **(A)** Pain, **(B)** neck disability index (NDI), **(C)** quality of life, and **(D)** follow-up effects on pain.

As an independent therapy, four studies reported the effects in relieving pain of TCEs for neck pain. All of them were included in the meta-analysis. The aggregated results indicated that TCEs alone showed better effects in improving pain (*SMD*, 0.81; 95% *CI*, 0.50–1.13; *p* < 0.00001, Figure 3A) compared with waiting list. But the aggregated results did not show better effects of TCEs alone in relieving pain compared with other exercises (*SMD*, 0.07; 95% *CI*, –0.18 to 0.33; *p* = 0.58, Figure 3A).

### Disability

As a complementary therapy, six studies reported the complementary effects in improving disability of TCEs for neck pain. Four of them were included in the meta-analysis. Baduanjin exercises showed beneficial effects in improving flexion (*SMD*, 0.65; 95% *CI*, 0.28–1.03; *p* = 0.0006, Figure 2B) and extension (*SMD*, 0.66; 95% *CI*, 0.12–1.19; *p* = 0.02, Figure 2B) of the neck. However, the aggregated result did not show that Baduanjin exercises had better complementary effects in improving disability (*SMD*, –0.05; 95% *CI*, –0.35 to 0.25; *p* = 0.73, Figure 2C).

As an independent therapy, the aggregated result showed beneficial effects of TCEs in improving disability of patients with neck pain (*SMD*, 0.74; 95% *CI*, 0.40–1.08; *p* < 0.0001, Figure 3B).

### Quality of Life

In the included studies, only three studies reported the effects of TCEs on quality of life in patients with neck pain. In the meta-analysis, the aggregated result did not show better effects of TCEs alone on quality of life in patients with neck pain (*SMD*, 0.03; 95% *CI*, –0.34 to 0.39; *p* = 0.89, Figure 3C). Hu et al. (2014) reported that 24-weeks TCEs showed potential effects in quality of life in patients with neck pain, especially for mental health.

### The Follow-Up Effects

In the follow-up effects, the aggregated results indicated that TCEs alone showed beneficial effects in relieving pain (*SMD*, 0.69; 95% *CI*, 0.39–1.00; *p* < 0.00001, Figure 3D). Feng and Qin (2017) reported that participants in Baduanjin group experienced less pain recurrence compared with massage group (7.14% vs. 20.83%) after 3 months follow-up.

### Publication Bias

**FIGURE 4:**
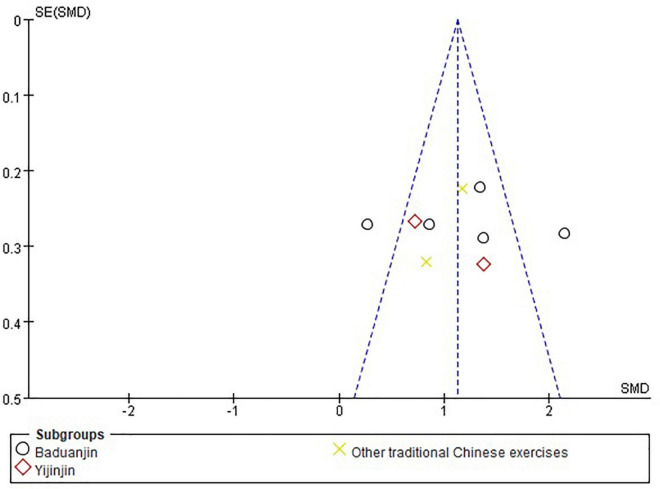
The funnel plots of the complementary effects of TCEs for neck pain in relieving pain.

The funnel plots for TCEs for neck pain in relieving pain including 9 RCTs, respectively, in Figure 4. The publication bias was small because the spots were substantially symmetric.

### Adverse Events

TCEs are considered to be safer movements, but there were some minor adverse events such as nausea, aching muscles, muscle tension, and falls and abrasions (von Trott et al., 2009; Lauche et al., 2016).

## Discussion

The current systematic review and meta-analysis were conducted to evaluate the effects of TCEs for neck pain. The peak prevalence of neck pain usually is occurred in the middle-aged and elderly populations; thus the average age of included participants was limited (more than 40 years). In most included studies, TCEs were used as a complementary therapy for neck pain. There are encouraging results suggesting that TCEs have complementary effects in improving pain of middle-aged and elderly patients with neck pain, especially Baduanjin exercises. Baduanjin exercises also showed beneficial effects in improving the range of cervical motion of middle-aged and elderly patients with neck pain. However, Baduanjin exercises did not show better complementary effects in improving disability of patients with neck pain, which may be related to fewer included studies. There was not enough evidence on the follow-up effects of TCEs for middle-aged and elderly patients with neck pain. As a complementary therapy, TCEs may provide more improvements in cervical balance, postural control, and blood circulation to relieve pain (Huston and McFarlane, 2016; Kuo et al., 2021). In addition, the middle-aged and elderly individuals could have similar health benefits through low to moderate-intensity TCEs, but experience lower metabolic energy.

As an independent therapy, the results supported the positive effects in relieving pain of TCEs alone compared with waiting list, but no significant differences were shown for effects of TCEs alone in relieving pain compared with other exercises. It indicates that TCEs may be similar as exercise therapy in relieving neck pain. Guidelines recommend exercise therapy as the first-line non-pharmacological therapy for neck pain (Bussières et al., 2016). Compared with common exercise therapy, TCEs have lower energy metabolism and similar health benefits. However, the mechanism of TCEs in the treatment of neck pain is not clear. They may also improve aerobic capacity and regulate the cervical balance of dynamic and static mechanics, so as to improve neck function, control posture, and relieve pain (Kuo et al., 2021). TCEs combined deep breathing with physical movements, and may have an influence on negative emotions. A study suggested that TCEs may reduce pain-related symptoms by improving cognitive appraisal outcomes such as anxiety and depression (Hall et al., 2016). Negative emotions usually originate from the chronic musculoskeletal pain. However, most included studies did not focus on mental health of patients with neck pain. Thus, TCEs did not show better effects in quality of life in middle-aged and elderly patients with neck pain. In our review, the aggregated result showed beneficial effects of TCEs in improving disability of middle-aged and elderly patients with neck pain, but it was based on the small sample size. In further studies, more high-quality evidence will be needed to support the effects of TCEs alone for middle-aged and elderly patients with neck pain.

The previous systematic review reported that there was insufficient evidence to support the clinical use of TCEs in improving pain intensity and enhancing functional mobility and quality of life in individuals with neck pain (Xie et al., 2021). However, there were only six studies of TCEs for neck pain published in English. TCEs were widely used for musculoskeletal pain in China; however, the studies published in China were excluded in their review. Therefore, there was a significant publication bias in their results. These Chinese studies of TCEs for neck pain were included in our review. TCEs usually were used as a complementary therapy for neck pain in China. In our review, the included studies used TCEs plus routine rehabilitation, acupuncture, Tuina, or medicine for neck pain. Therefore, the complementary effects and independent effects of TCEs for neck pain were evaluated separately in our review. In addition, the subgroup analysis was conducted based on different TCEs in our meta-analysis. The previous systematic review also showed similar results with our review. Li et al. (2021) reported that TCEs could effectively relieve the clinical symptoms of patients with musculoskeletal pain. Therefore, there was more powerful evidence of TCEs for neck pain in our review.

### Limitation

There are several limitations in this review. First, a rigorous search strategy was applied in our review, but there may be some uncertainty bias in location and publication (Moncrieff et al., 1998). Second, only RCTs were included in our review, but it was impossible to blind the patients and therapists in the studies of TCEs. The blinded outcome assessors and concealed allocations could partly compensate for these flaws, but only two trials used concealed allocation and four studies blinded the outcome assessors. Third, the subgroup analysis based on different TCEs was only conducted on the complementary effects in relieving pain. In other outcomes, detailed subgroup analysis was not performed due to insufficient eligible studies. Forth, our results may be affected by the different style, frequency, duration, and session of TCEs. Further reviews should focus on the parameters of TCEs if there are enough eligible studies. Fifth, the result of this review may be influenced by the type of neck pain, but the subgroup analysis could not be conducted due to few included studies. However, it is certain that the neck pain originates from the cervical degenerative diseases in most included studies. Finally, it was suspicious that most included studies conducted in China had non-participants dropout.

## Conclusion

The current systematic review demonstrated that there was the positive evidence to support the clinical use of TCEs, as a complementary therapy, in relieving pain of middle-aged and elderly patients with neck pain, especially Baduanjin exercises. However, the evidence supporting the effects of TCEs alone in improving pain and disability of middle-aged and elderly patients with neck pain was limited due to the small sample size. The follow-up effects of TCEs were still insufficient.

## Data Availability Statement

The original contributions presented in the study are included in the article/supplementary material, further inquiries can be directed to the corresponding author/s.

## Author Contributions

LK, JR, XZ, and MF conserved and designed the study. TH and JR performed the literature search. JR and SF identified and selected the studies. LK and XZ assessed the methodological quality and extracted data. LK, XZ, and MF performed data synthesis and analysis. LK, JR, SF, XZ, and MF wrote the draft manuscript. All authors contributed to the article and approved the submitted version.

## Conflict of Interest

The authors declare that the research was conducted in the absence of any commercial or financial relationships that could be construed as a potential conflict of interest.

## Publisher’s Note

All claims expressed in this article are solely those of the authors and do not necessarily represent those of their affiliated organizations, or those of the publisher, the editors and the reviewers. Any product that may be evaluated in this article, or claim that may be made by its manufacturer, is not guaranteed or endorsed by the publisher.
